# Fungal glycans and the innate immune recognition

**DOI:** 10.3389/fcimb.2014.00145

**Published:** 2014-10-14

**Authors:** Eliana Barreto-Bergter, Rodrigo T. Figueiredo

**Affiliations:** ^1^Departamento de Microbiologia Geral, Instituto de Microbiologia, Universidade Federal do Rio de JaneiroRio de Janeiro, Brazil; ^2^Instituto de Ciências Biomédicas/Unidade de Xerém, Universidade Federal do Rio de JaneiroRio de Janeiro, Brazil

**Keywords:** fungal pathogens, polysaccharides, glycoconjugates, pattern recognition receptors, innate immunity

## Abstract

Polysaccharides such as α- and β-glucans, chitin, and glycoproteins extensively modified with both *N*- and *O*-linked carbohydrates are the major components of fungal surfaces. The fungal cell wall is an excellent target for the action of antifungal agents, since most of its components are absent from mammalian cells. Recognition of these carbohydrate-containing molecules by the innate immune system triggers inflammatory responses and activation of microbicidal mechanisms by leukocytes. This review will discuss the structure of surface fungal glycoconjugates and polysaccharides and their recognition by innate immune receptors.

## Introduction

The fungal cell wall is basically comprised of chitin and β-glucans, which form an inner rigid core, but several other structurally complex polysaccharides, glycoproteins, enzymes, and lipids have been identified as cell wall components, frequently loosely anchored in the external layer. The fungal cell wall is a dynamic structure where its polymeric constituents are under continuous chemical modification and refolding during their biosynthesis. The cell wall is an excellent target for the action of antifungal agents and also a target for the innate immune recognition, since most of its components are absent from mammalian cells (Masuoka, 2004; Latgé, 2010).

## Fungal glycoconjugates and polysaccharides

### Mannans

The cells of the majority of yeasts contain mannose-containing polysaccharides that are often covalently attached to protein. These polysaccharides are predominantly α-D-manopyranans that display a variety of sequences of linkage types in branched structures. A few of the branched mannans carry some β-D-manopyranose residues in the side chains (Gorin et al., 1969). In phosphomannans some sugar residues are attached through phosphorodiester linkages (Masuoka, 2004). The cell surface of *Candida* species is surrounded by a layer enriched in mannosylated glycoproteins and mannosylated lipids. These cell wall mannoproteins (CWMPs) are non-covalently bound to the cell wall as phosphopeptidomannan (PPM) or covalently attached to β (1 → 6) glucan through remnant glycosylphosphatidylinositol (GPI) anchors (GPI-anchored proteins), which is in turn attached to β (1 → 3) glucan or chitin, that form the inner cell wall layer (Masuoka, 2004). PPM containing *O*- and *N*-linked oligosaccharides, can be obtained by autoclaving yeast cells. *O*-linked mannose residues associated with serine/threonine consist of short chains of α-(1 → 2) or α-(1 → 3)-linked mannose. *N*-linked mannans consist of an inner core elongated by an α-(1 → 6)-linear chain with branched side chains of α-(1 → 2) or α-(1 → 3) mannose (Shibata et al., 2003). This structure is similar to that of *Saccharomyces cerevisiae*, extensively investigated (Stewart and Ballou, 1968). In addition, a number of yeast mannans contain β-D-mannopyranosyl units in the side chains. β-(1 → 2)-linked mannose residue is also present in the CWMP of some *Candida* species. *C. albicans* serotype A, *C. glabratta*, *C. tropicalis*, and *C. lusitanea* have the antigenic factor 6, which corresponds to the Man β (1 → 2)-Manα(1 → 2) Manα 1 residue (Kobayashi et al., 1992).

A cell wall lipoglycan, phospholipomannan (PLM) is another glycoconjugate of *C. albicans* that presents β-mannosides. It consists of linear chains of β-(1 → 2)-Man*p* units and inositol covalently linked through a phosphodiester bond to a lipid moiety (Trinel et al., 2002). The degree of polymerization of β-(1 → 2) mannosides chains is up to 19 in *C. albicans* serotype A strains and short β-(1 → 2) mannosides chains with predominance of mannotriose in *C. albicans* serotype B (Trinel et al., 2005). PLM is able to activate inflammasome pathway through a ROS-independent mechanism and this activity seems to be related with the lipid moiety of the molecule. On the other hand, the induction of TNF-α production is dependent on the glycan moiety (Devillers et al., 2013). Long glycan chains in PLM favors the formation of complexes with a glycan-binding protein, galectin-3, secreted by macrophages (Fradin et al., 2000).

The role of mannosylation has been extensively investigated in glycosylation deficient mutants. The Ca^+2^/Mn^+2^ ATPase Pmr1p is required for the transport of Mn^+2^ the Golgi apparatus, where it is necessary as a cofactor for the activity of mannosyltransferases in *S. cerevisiae* and *C. albicans*. The *pmr1* deficient *C. albicans* strain shows a strong reduction of the mannose content in the cell wall which reflects a great reduction in the *N*- and *O*-linked glycosylation and phosphomannan synthesis. The *pmr1* deficient *C. albicans* also exhibits an increased susceptibility to cell wall noxious agents, and interestingly this strain shows a constitutive activation of Mkc1p, a MAP kinase involved in the activation of signaling pathways required for the maintenance of the cell wall integrity (Bates et al., 2005). The phenotype of the *pmrp1* deficient *C. albicans* is similar to that observed in the *och1* deficient *C. albicans*, that lacks the 1,6-mannosyltransferase Och1p and, as a consequence, this strain does not form the α1,6-linked polymannose core, while the *O*-glycosylation remains functional (Bates et al., 2006). The double deficient *mnt1/mnt2 C. albicans* strain lacks the activity of the α-1,2-mannosyltransferases, Mnt1p and Mnt2p, what results in the absence of the Man_2_–Man_5_ residues, and therefore in the absence of *O*-linked glycosylation. As observed in the *C. albicans* mutant strains that lack the *N*-linked glycosylation, the *mnt1/mnt2* deficient strain shows an increased susceptibility to cell wall damaging agents (Munro et al., 2005).

Besides its role in the cell wall architecture, the mannosylation plays an important role in the growth and morphology of *C. albicans*. Although, mannosylation deficient *C. albicans* strains have been demonstrated to grow *in vitro*, they present morphological alterations with a deficient filamentation and formation of aggregates by the yeasts (Bates et al., 2005, 2006; Munro et al., 2005). Thus, an adequate mannosylation plays an important role in the *C. albicans* cell wall integrity and development of the morphological stages of *C. albicans*. Interestingly, while *N*-glycosylation and *O*-glycosylation are required for the cell wall protection against environmental stress, as well as for the development of *C. albicans*, the synthesis of phosphomannans is dispensable for these aspects, since the selective phosphomannan deficient strain, *mmn4*, exhibits a similar growth, morphology, and susceptibility to cell wall toxic agents (Hobson et al., 2004).

Mannosylation plays a critical role in the virulence of *C. albicans*. *N*- and *O*-linked mannans are required for the virulence of *C. albicans* in model of systemic candidiasis, while the presence of phosphomannans is dispensable (Hobson et al., 2004; Bates et al., 2005, 2006; Munro et al., 2005). Although the *N*- and *O*-glycosylation deficient *C. albicans* strains present a decreased lethality in experimental models of infection, a deficient *N*-glycosylation does not impact the fungal loads during the infection. In contrast, the *O*-glycosylation deficient *C. albicans* strain presents decreased fungal counts in the experimental infection, suggesting that while *N*- and *O*-glycosylation are required for the virulence, only *O*-glycosylation is necessary for the colonization of the organs during the systemic infections (Munro et al., 2005; Bates et al., 2006). Interestingly, the *O*-glycosylation deficient *C. albicans* strain (*mnt1*Δ/*mnt2*Δ) shows a deficiency in the adhesion to collagen matrices and epithelial cells, while *N*-glycosylation, but nor *O*-glycosylation, is required for the phagocytosis, binding and cytokine production by macrophages and dendritic cells, suggesting that *O*-linked mannans are involved in the tissue colonization, while *N*-linked mannans are major determinants of the innate immune recognition (Munro et al., 2005; Bates et al., 2006; Cambi et al., 2008). The impact of the glycosylation pathways involved in the mannosylation of fungal glycoconjugates in the biology and virulence of other pathogenic fungi is largely unknown. However, since the extensive mannosylation of glyconconjugates is a conserved pattern found in fungi, the distinct patterns of glycosylation must be determinants for the fungal pathogenesis. Thus, genetic approaches targeting glycosylation pathways in pathogenic fungi must be useful for the comprehension of the fungal virulence and biology.

### Heteropolysaccharides with mannan main chains

Peptidorhamnomannans (PRMs) are common cell wall components that are distributed in species of the *Scedosporium*/*Pseudallescheria* complex and can be isolated by the methodology routinely used in our laboratory (Lopes et al., 2011). Hot aqueous extraction, followed by treatment with Cetavlon in the presence of sodium borate, provided a precipitate of peptidorhamnomannan (PRM), containing carbohydrate *N*- and *O*-linked to peptide. Methylation-gas chromatography-mass spectrometry (GC-MS) analysis and ^1^H- and ^13^C-nuclear magnetic resonance (NMR) spectroscopy of PRM showed to contain α-Rha*p*-(1 → 3)-α-Rha*p*-side-chain epitopes linked (1 → 3) to a (1 → 6)-linked α-Man*p* core (Pinto et al., 2001). Non-reducing, *O*-linked oligosaccharides were isolated from the PRMs of *P. boydii*, *S. apiospermum*, and *S. prolificans* mycelium by alkaline β-elimination under reducing conditions (Pinto et al., 2005; Barreto-Bergter et al., 2008, 2011). Three major oligosaccharides were obtained and their structures elucidated based on a combination of techniques including gas chromatography, Electrospray Ionization Mass Spectrometry (ESI-MS), ^1^H (obs), ^13^C Heteronuclear Multiple-Quantum Correlation-NMR (HMQC-NMR) spectroscopy and methylation analysis (Pinto et al., 2005). It is interesting to note that these different carbohydrate epitopes present an conserved α-Rha*p*-(1 → 3)-α-Man*p*-(1 → 2)-α-Man*p*-(1 → structural component. PRM *O*-linked oligosaccharides were also isolated from *Sporothrix schenckii* (Lopes-Bezerra, 2011). Besides glucuronic acid–containing oligosaccharides, a trisaccharide α-Rha*p* (1 → 3)-Man*p* (1 → 2)-Man-ol similar to a conserved structural component of the *Scedosporium*/*Pseudallescheria* complex was identified (Lopes-Bezerra, 2011). PRMs are antigenic and *O*-linked glycans may account for a significant part of PRM antigenicity, since de-*O*-glycosylation treatment has been demonstrated to decrease its antigenicity by 70–80% (Pinto et al., 2005). Similar results were obtained with *A. fumigatus* PGM (Leitao et al., 2003) and PRM from *S. schenckii* (Lopes-Bezerra, 2011). The immunodominance of the *O*-linked oligosaccharide chains was evaluated by testing their ability to inhibit reactivity between PRM and anti-*P. boydii* antiserum in an ELISA hapten system (Pinto et al., 2005). Up to 75% inhibition occurred with both penta- and hexasaccharides from *P. boydii* PRM. Similar results were observed using penta- and hexasaccharides from *S. prolificans* (our unpublished results). These oligosaccharide alditols blocked recognition between rabbit sera and intact PRM in a dose-dependent manner. Thus, *O*-linked oligosaccharide chains, despite being the less abundant carbohydrate components of the *P. boydii* and *S. prolificans* glycocomplexes, may account for a significant part of the antigenicity associated with the rhamnomannan component of *P. boydii/S. prolificans*. Rhamnomannans were also isolated from *P. boydii* using a hot alkaline extraction and their structures determined by one-dimensional (1H and 13C) and two-dimensional Correlation Spectroscopy (COSY), Total Correlation Spectroscopy (TOCSY), and Heteronuclear Single Quantum Correlation (HSQC) experiments. The NMR data of fraction II showed at C-1 signals at δ 97.9/4.981, 101.0/4.967, 102.2/5.228, and 103.9/5.060, typical of terminal α-rhamnose units, *O*-3,6-substituted- α-mannopyranose, O-2-substituted- α-mannopyranose and α-Man*p*-3-O-substituted units, respectively. The signal at δ 79.9/4.127 confirms the 3-O-substituted α-Man*p* units (Figueiredo et al., 2010).

PRMs derived from *P. boydii* are also involved in the adhesion and infection of an epithelial cell line by *P. boydii* conidia, since the competition with soluble PRM and anti-PRM antibodies are able to inhibit the entry of conidia. Interestingly the determinants involved in the interactions mediated by PRMs seem to require the presence of terminal rhamnose residues that are eliminated by partial hydrolysis, while *O*-glycosylation, the protein portion or mannosyl residues are not necessary (Pinto et al., 2004). *P. boydii* derived rhamnomannans induce cytokine release by macrophages in a mechanism dependent on TLR4 signaling, as well as degradation of IkBα and phosphorylation of MAPKs. The induction of cytokine production by these molecules requires the presence of terminal non-reducing residues of rhamnose, since their removal by partial acid hydrolysis abolishes the ability of rhamnomannan to induce cytokine production by macrophages. Thus, structures with terminal rhamnose and mannose residues present in *P. boydii* rhamnomannans are likely structural motifs involved in TLR recognition (Figueiredo et al., 2010). Thus, rhamnomannans represent a characteristic pattern of glycosylation found on the surface of fungi of the complex *Scedosporium/Pseudallescheria*, mediating cell infection and the innate immune recognition.

Galactomannans are important structural components of the *Aspergillus* cell-wall, being widely distributed among most *Aspergillus* species. Galactomannans are mannose-containing polysaccharides containing terminal D-galactose residues in both furanose and pyranose ring forms, and for the latter both α-D and β-D configurations have been encountered (Latgé et al., 1994). A galactomannan was isolated either from a culture filtrate (Latgé et al., 1994) or from the fungal cell wall of *A. fumigatus* (Leitao et al., 2003). Its structure was elucidated by acid and enzymatic hydrolysis, partial acetolysis, methylation analysis, and ^13^C NMR spectroscopy (Leitao et al., 2003). It consists of a main chain of (1 → 6)-linked α-D-mannopyranosyl residues substituted at *O*-2 by 1–3 consecutive α-D-mannopyranosyl units that were (1 → 2)-linked. β-D-Galactofuranosyl-containing side-chains, with (1 → 5)-links and an average length of approximately 6 units, were attached to *O*-6 of the mannan core, β-D-Gal*f*-(1 → 5)-[β-D-Gal*f*-(1 → 5)]_0−5_-(1 → 6)-α-Man*p*-. Galactomannans have also been shown to exist in a glycosylinositol membrane-bound form. Chemical and enzymatic degradation and mass spectrometry analysis showed that the lipid anchor was a glycosylphosphatidylinositol (GPI), containing a C18-phytosphingosine and a monohydroxylated lignoceric acid, in the lipid portion (Costachel et al., 2005). Summarizing, galactomannans from *A. fumigatus* are found in three different forms, namely: (1) as a free polysaccharide found in the culture medium, (2) covalently linked to the β (1 → 3) glucans of the fungal cell wall, and (3) GPI-anchored to the membrane.

Besides the polysaccharides described above that are covalently interlinked to form a skeletal structure, *N*- and *O*-linked peptidogalactomannans were present in the outer layer of *A. fumigatus* cell wall and isolated by hot buffered aqueous extraction (Haido et al., 1998). *O*-linked oligosaccharides were selectively released from pGM by β-elimination under mild alkaline reductive conditions in the presence of sodium borohydride. Their primary structures were determined based on a combination of techniques including gas chromatography, ESI-QTOF-(Quadrupole followed by a time-of-flight mass analyzer) MS, ^1^H- and TOCSY, and ^1^H-^13^C HMQC NMR spectroscopy and methylation analysis, to be: α-Glc*p*-(1 → 6)-Man-ol, β-Gal*f*-(1 → 6)-α-Man*p*-(1 → 6)-Man-ol, β-Gal*f*-(1 → 5)-β-Gal*f*-(1 → 6)-α-Man*p*-(1 → 6)-Man-ol and β-Gal*f*-(1 → 5)-[β-Gal*f*-(1 → 5)]_3_-β-Gal*f*-(1 → 6)-Man-ol (Leitao et al., 2003). These *O*-linked oligosaccharides may account for a significant part of the peptidogalactomannan antigenicity, because de-*O*-glycosylation decreased by 50% its activity. The immunodominant epitopes were present in the tetra- and hexasaccharide, which contain a β-Gal*f*-(1 → 5)-β-Gal*f* terminal group (Leitao et al., 2003).

Another cell wall polysaccharide, a phosphonogalactomannan, was isolated via alkaline extraction from *A. versicolor* mycelia and its complex structure was identified by ^31^P, ^1^H, and ^13^C NMR spectroscopy and methylation analysis and appeared to have similar structure as the galactomannan from *A. fumigatus* and *A. niger*, except for the presence of phosphorodiester groups (Tischer et al., 2002).

Galactomannans are major antigens produced during the infections caused by *A. fumigatus*. Galactomannans are detected in the serum and bronchoalveolar lavage of patients with invasive aspergillosis, and their detection has been used as a diagnostic marker for the infections caused by *Aspergillus spp* (Acosta et al., 2011; He et al., 2012; Teering et al., 2014). Galactomannans are secreted by *A. fumigatus* and, in association with galactosaminogalactans, are major components of the biofilm produced by the *A. fumigatus* mycelium. Interestingly, the *A. fumigatus* biofilm is found around the mycelia in aspergilomas and pulmonary lesions found in invasive infections, indicating that this structure must participate in the pathogenesis of the infections (Loussert et al., 2010). Galactomannans are able to inhibit the *A. fumigatus* conidial phagocytosis by dendritic cells, indicating that the recognition of cell surface expressed galactomannans is required for the *A. fumigatus* binding and internalization (Serrano-Gómez et al., 2004). Thus, glycoconjugates containing galactomannans must represent important antigens and targets for the immune response, besides playing a role in the pathogenesis of aspergillosis, possibly acting as components of extracellular adhesive structures during the host tissue colonization.

### α-D-linked glucans

Another important group of polysaccharides, the α-glucans, have been isolated from several fungal cells. The fungal α-glucans described include α (1 → 3)-linked, and in some species glycogen-like α (1 → 4) and (1 → 6)-linked chains. The fungal α-glucans described show an outermost localization in the cell wall and are easily extracted by hot extraction, being soluble in alkaline conditions, in opposition to the largely insoluble β-glucans that form the rigid core of the cell wall.

Pseudonigeran, isolated following alkaline extraction of cell walls of *A. niger*, is an α (1 → 3)-linked α-D-glucopyranan as shown by methylation, periodate oxidation, and partial hydrolysis studies (Horisberger et al., 1972). It is also present in cell walls of *A. nidulans*, *A. fumigatus*, and *Cryptococcus* spp (Bacon et al., 1968; Zonneveld, 1972). An α (1 → 3)-glucan is present in the outer most layer of the *Histoplasma capsulatum* yeast cell wall (Rappleye et al., 2007), whereas the mycelial form contains none (Kanetsuna et al., 1974). In *Blastomyces dermatiditis* and *Paracocciodioides brasiliensis* the levels of the α (1 → 3)-glucans are much higher in the yeast than in the mycelial form (Kanetsuna and Carbonell, 1970, 1971; Kanetsuna et al., 1972). Another α-glucan has been isolated from *P. boydii* and its structure was determined, using a combination of techniques including gas chromatography, ^1^H TOCSY, ^1^H, and ^13^C NMR spectroscopy and methylation analysis, to be a glycogen-like polysaccharide consisting of linear 4-linked α-D-Glc*p* residues substituted at position 6 with α-D-Glc*p* branches (Bittencourt et al., 2006). A similar structure was detected in *A. fumigatus* (Bahia et al., 1997).

The role of α-glucans in the fungal biology is still incompletely understood. During the germination of *A. fumigatus* conidia, the exposure of α (1 → 3)-glucans promotes the aggregation and development of the germ tubes (Fontaine et al., 2010). In *H. capsulatum*, α (1 → 3) glucans mask the β (1 → 3) glucans what results in a deficient recognition of these molecules by Dectin-1, and this has been speculated to avoid the induction of pro-inflammatory cytokines by macrophages (Rappleye et al., 2007). Glycogen-like α-glucans isolated of *P. boydii* are able to inhibit the phagocytosis of *P. boydii* conidia, furthermore these molecules induce the release of pro-inflammatory cytokines by murine macrophages, thus indicating that α (1 → 4)(1 → 6) glucans represent immunostimulatory molecules mediating the recognition of *P. boydii* by macrophages (Bittencourt et al., 2006). Thus, the investigation of the roles of α-glucans in the fungal development and interaction with immune cells must bring important insights in the fungal biology and virulence.

### β-D-linked glucans

β-D-Glucans are on interest because of their potential in modulating a wide range of innate host immune responses. They are present in virtually all fungi. A number of studies carried out on fungal polysaccharides have been demonstrating the presence of predominant 3-linked β-D-glucopyranosyl structures. A linear cell wall glucan from *S. schenckii* contains 3-*O*-, 6-*O*-, and 4-*O*-substituted β-D-glucopyranosyl units (Previato et al., 1979). Cell-wall polysaccharides, such as β (1 → 3)-glucans, have been characterized in *Aspergillus* spp (Bernard and Latgé, 2001). The β-D-glucopyranan from the mycelial form of *P. brasiliensis* contains 90% of (1 → 3) linkages. This polymer is a major constituent of the cell wall of the filamentous phase of *H. capsulatum* (Davis et al., 1977). The β-D-glucopyranans of *C. albicans* serotype B and *C. parapsilosis* are mainly linear, with only approximately 10% of branch points, and contain, principally, (1 → 6) linkages (67 and 63%, respectively) (Yu et al., 1967). *C. albicans* does not contain α-glucans. It only contains both β-1,3 and β-1,6-glucans, but no mixed intrachain β-1,3/1,6 linkages. Analysis by proton NMR spectroscopy (NMR) of glucans from yeast or hyphal forms of *C. albicans* showed that they were different from *S. cerevisiae* glucans in side-chain branching and reducing termini (Ruiz-Herrera et al., 2006).

In a recent work, Lowman et al. (2014) using a mild extraction procedure for isolation of *Candida albicans* yeast and hyphal forms showed by NMR and GC-MS analysis that the hyphal glucan has a unique cyclic structure, not found in yeast glucan. Both are branched glucans having a (1 → 3)-linked, β-D-glucopyranosyl main-chain, partially substituted at *O*-6 by (single unit) β-D-glucopyranosyl groups. However, in addition to these linkages, a 2,3 linkage was identified and this feature has not been reported previously in *C. albicans* (Lowman et al., 2014). Interestingly, the cyclic hyphal β-glucan presents a differential pattern of cytokine induction by human monocytes in relation to that promoted by linear yeast β-glucans, with cyclic β-glucans being a more potent inducer of IL-1β, TNF, and IL-6 than the linear yeast β-glucans. As observed for the linear β (1 → 3) glucans the cyclic hyphal β-glucans are recognized by Dectin-1 (Lowman et al., 2014).

Fungal β-glucans have been recognized for so long as immunomodulators (Goodridge et al., 2009b). Large β-glucan particles induce cytokine production, reactive oxygen species (ROS) production and phagocytosis by neutrophils and macrophages, while soluble β-glucans act as antagonists of these responses (Brown and Gordon, 2001; Brown et al., 2003; Gantner et al., 2003; Kennedy et al., 2007; Goodridge et al., 2011). Furthermore, β-glucans induce the activation of the alternative pathway of the complement cascade (Bose et al., 2013). β-glucans are also efficient adjuvants, promoting dendritic cell maturation and antigen loading by these cells, what triggers the activation of CD4 and cross-priming of CD8 lymphocytes (Yoshitomi et al., 2005; Leibundgut-Landmann et al., 2008; Weck et al., 2008).

Recognition of fungal β-glucans by macrophages requires the exposure of the inner layer of the fungal cell wall, as a result of the growth or germination, as observed during division and septation of *C. albicans* yeasts and hyphae, and germination of resting conidia of *A. fumigatus* (Gantner et al., 2005; Hohl et al., 2005; Steele et al., 2005; Gersuk et al., 2006). Thus, the β-glucan recognition must represent a mechanism for the detection of the growth and morphological differentiation of pathogenic fungi. Interestingly, *A. fumigatus* and other environmental fungi express a highly hydrophobic external layer formed by hydrophobin proteins which masks the β-glucans in the resting conidia, and it has been speculated that the recognition of exposed β-glucans in the germinating conidia, but not in the resting ones, could have evolved to avoid the persistent inflammation, to the ubiquitous resting conidia, while permitting the detection of the invasive morphological stages (germ tubes and hyphae) (Aimanianda et al., 2009).

### Chitin

Chitin is an important skeletal component in most fungi. Chitin is a linear polysaccharide composed by 4-linked-2-acetamido-2-deoxy-β-D-glucopyranan (Munro and Gow, 2001). Chitin and β-glucans are the most abundant polysaccharides conserved through the evolution in the fungal cell wall and are the most common polysaccharides in fungal species. Chitin represents a small percentage in *S. cerevisiae*, but the content is higher in other yeasts and filamentous fungi (Xie and Lipke, 2010). Chitin composes the insoluble core of the fungal cell wall, either isolated or associated with β-glucans (Masuoka, 2004). The composition of the alkali insoluble core of the *A. fumigatus* cell wall has been described, and it contains pure chitin chains, as well as chitin chains associated to β-1,3 glucans and chitin/galactomannans associated to the side branching chains of β-1,3 glucans (Fontaine et al., 2000).

Chitin and chitosan, its deacetylated polymer, present several immunomodulatory effects. Exposition to chitin has been implicated in the development of allergic airway inflammation, and in an experimental model of pulmonary inflammation induced by *Aspergillus* cell wall, the digestion with chitinase, decreases the inflammation, and leukocyte recruitment (Van Dyken et al., 2011). Chitin induces cytokine production, leukocyte recruitment, and alternative activation of macrophages (Reese et al., 2007; Da Silva et al., 2009, 2010). Furthermore, macrophages promptly ingest chitin and chitosan particles (Nishiyama et al., 2006). Curiously, chitosan, but not chitin, is able to promote the activation of the inflammasome NLRP3/ASC/caspase-1, and therefore chitosan leads to the activation of caspase-1 and release of IL-1β (Bueter et al., 2011, 2014).

### Galactosaminogalactan

Galactosamine-containing polysaccharides have been identified in *Aspergillus* species. An exocellular polysaccharide from *A. nidulans* is a linear molecule and according to methylation analysis contains 4-*O*-substituted α-D-galactopyranosyl and 4-*O*-substituted 2-acetamido-2-deoxy-α-D-galactopyranosyl units. Periodate oxidation and ^1^H-NMR data showed their ratio to be approximately 1.8:1.0. (Gorin and Eveleigh, 1970). A heteropolysaccharide from *A. niger* has a related structure but lacks *N*-acetyl groups. It contains 4-*O*-substituted α-D-galactopyranosyl and 2-amino-2-deoxy-α-D-galactopyranosyl units in the ratio 7:2 (Bardalaye and Nordin, 1976, 1977). A galactosaminogalactan secreted by the mycelium of *A. fumigatus* was identified and the carbohydrate structure analysis showed that it is a linear heterogeneous polymer of α-1-4 galactosyl and α1-4 *N*-acetylgalactosaminyl residues (Fontaine et al., 2011). Galactosaminogalactans have been localized on the outer layer of the *A. fumigatus* cell wall and due to its localization might mask the exposure of other polysaccharides such as β-(1 → 3)-glucan (Gravelat et al., 2013). *In vitro* studies suggest that this molecule is the principal mediator of *A. fumigatus* adherence and plays a role in biofilm formation (Loussert et al., 2010; Gravelat et al., 2013). The *A. fumigatus* galactosaminogalactans are antigenic, and anti-galactosaminogalactans antibodies are present in the human serum, even in the absence of *Aspergillus* spp infections, but these polysaccharides are in fact immunosuppressive, as observed by decreased neutrophil recruitment, and higher fungal loads, during the *A. fumigatus* experimental infection following the immunization with galactosaminogalactans (Fontaine et al., 2011). The immunosuppressive properties of *A. fumigatus* galactosaminogalactans have been attributed to the inhibition of the production of IFN-γ, neutrophil chemoattractant chemokines, and the induction of IL-1Ra, an antagonist cytokine for the IL-1 receptor (Gresnigt et al., 2014).

## Pattern recognition receptors involved in the recognition of fungal carbohydrates

### Toll like receptors (TLRs)

TLRs comprise a family of receptors that share homology with the Toll receptor (Takeuchi and Akira, 2010). The Drosophila Toll was described as a regulator of the dorsoventral differentiation in the embryo of *Drosophila*, and further pointed as a receptor required for the antifungal immune responses in *Drosophila* (Lemaitre et al., 1996). Following the characterization of the Toll receptor in *Drosophila*, 10 human TLRs, and 13 murine TLRs have been described (Takeuchi and Akira, 2010; Hidmark et al., 2012; Li and Chen, 2012; Oldenburg et al., 2012). TLRs present an amino-terminal extracellular domain containing leucine rich repeats and intracellular domains that share homology with the Toll/IL-1 receptor (TIR) domain. The TIR domains recruit adaptor proteins containing TIR domains, such as MyD88, TRIF, TRAM, and TIRAP, what leads to signaling pathways that culminate in the activation of the transcriptional complexes NF-κB, AP-1, IRFs (IRF3/7), and MAP kinases, and as consequence in the expression of cytokines and co-stimulatory molecules (Takeuchi and Akira, 2010).

TLR4 is the receptor responsible for responses to the bacterial lipopolysaccharides (LPS) (Poltorak et al., 1998). The LPS recognition by TLR4 requires the association with MD2 (Shimazu et al., 1999; Schromm et al., 2001; Akashi et al., 2003). TLR4 has been demonstrated to recognize fungal mannans. *S. cerevisiae* and *C. albicans* derived mannans induce cytokine production by human monocytes by a mechanism dependent on CD14, TLR4 and this is amplified in the presence of the Lipopolysaccharide Binding Protein (LBP) (Tada et al., 2002). Netea et al. reported, in an extensive investigation of mannosylation-defective *C. albicans* strains, that TLR4 cooperates with the Mannose Receptor (MR) in the recognition of mannans, with TLR4 detecting *O*-linked mannans, while MR is responsible for the sensing of *N*-linked mannans (Netea et al., 2006). TLR4 is also involved in the recognition of PRMs isolated from *P. boydii* (Figueiredo et al., 2010) and in detection of the glucuronoxylomannans from *C. neoformans* (Shoham et al., 2001), indicating that TLR4 acts as a receptor for the sensing of distinct mannose containing polysaccharides.

TLR2 recognizes bacterial lipoproteins and lipoteichoic acid, mycobacterial lipoarabinomannans, and GPI anchors from protozoan parasites (Takeuchi et al., 2000; Campos et al., 2001; Sandor et al., 2003; Tapping and Tobias, 2003; Krishnegowda et al., 2005). The recognition mediated by TLR2 have been described to involve the dimerization with TLR1, for the detection of triacylated lipoproteins (Takeuchi et al., 2002; Jin et al., 2007), or TLR6, in case of diacylated lipoproteins (Takeuchi et al., 2001; Kang et al., 2009).

TLR2 has also been pointed as a receptor involved in the recognition of fungal molecules. TLR2 is the receptor responsible for the activation of NF-κB and cytokine release by macrophages in response to a PLM isolated from *C. albicans*, while TLR4 and TLR6 contribute partially to the responses evoked by this lipoglycan (Jouault et al., 2003). TLR2 has also been described as a receptor involved in the recognition of glucogen-like α-1,6-branched α-1,4-glucans, such as an enzymatically produced glycogen (Kakutani et al., 2012), and a glycogen-like α-glucan purified from *P. boydii* (Bittencourt et al., 2006). TLR2/TLR1 and TLR2/TLR6 is also involved in the recognition of the glucuronoxylomannans isolated from *C. neoformans* and *C. gatii* capsules (Fonseca et al., 2010).

The mechanisms by which TLR4 and TLR2 recognize mannose-containing glycoconjugates and other fungal polysaccharides are poorly understood. TLR4 and TLR2 have been demonstrated to bind fungal polysaccharides, and these interactions are inhibited in the presence of soluble mannans and fucose (Hsu et al., 2009). Mannans occur as polysaccharide components in the structures of many fungal glycoconjugates, such as glycoproteins and glycolipids (Masuoka, 2004). In this way, the widely employed mannan preparations are in fact highly heterogeneous mixtures of mannosylated glycoproteins, and possibly glycolipids. Thus, the use of purified mannans, as activators, ligands or competitors for TLR4 or TLR2 mediated responses, although useful in demonstrating the recognition of these fungal glycoconjugates, fails in elucidating the specific structural motifs involved in the activation of these receptors.

The structural bases for the recognition of bacterial lipoproteins and LPS, by TLR2/TLR1 and TLR2/TLR6, and TLR4/MD2, respectively, have been elucidated by crystallographic analyses (Jin et al., 2007; Kang et al., 2009; Park et al., 2009). The emerging pattern indicates that fatty acid chains in the bacterial ligands interact with hydrophobic pockets in the extracellular domain localized in the interfaces of the receptor complexes. It is unclear how TLR2 and TLR4 recognize hydrophilic ligands such as polysaccharides, but it seems reasonable that the interaction of these receptors with carbohydrates must work in a distinct way from the classical bacterial lipid ligands. In this sense, the role of TLR2 and TLR4 as receptors have been extensively expanded to the recognition of several distinct molecules, including heme (Figueiredo et al., 2007), hyaluronic acid (Termeer et al., 2002; Jiang et al., 2005; Scheibner et al., 2006), heparan sulfate (Johnson et al., 2002; Brunn et al., 2005) and biglycan (Schaefer et al., 2005). Thus, the mechanisms of recognition by these receptors seem to be wider than the previously described recognition of microbial lipid molecules.

In conclusion, it seems evident that more detailed investigations are required for the characterization of the mechanisms involved in the recognition of fungal carbohydrates by TLR2 and TLR4. Alternative approaches such as, (1) use of chemically defined oligosaccharides as ligands for TLR2 and TLR4, (2) specific enzymatic digestion of fungal polysaccharide preparations and analysis of TLR2 and TLR4 activation/binding, (3) extensive purification and elucidation of the structures of fungal polysaccharides by complementary analytic tools (mass spectrometry, RMN) and co-relation with TLR2 and TLR4 activation, and (4) TLR2 and TLR4 binding assays must be determinant for the elucidation of the recognition of fungal polysaccharides by TLR2 and TLR4.

### C-type lectin receptors (CLR)

CLR comprise an important group of proteins involved in the recognition of fungal pathogens. CLR are defined by the presence of domains composed by two loops joined by disulfide bonds, the C-type Lectin Domains (CTLDs), which contain carbohydrate recognition domains (CRD). CLR are characterized by the interaction with carbohydrates that in many cases requires the presence of Ca^+2^ (Sancho and Reis E Sousa, 2012). Two conserved motifs are present in the CRD and dictate the specificity for carbohydrates; the EPN motif confers binding to mannose, *N*-acetylglucosamine, L-fucose, and glucose, while a QPD motif determines the recognition of galactose and *N*-acetylgalactosamine (Drickamer, 1992; Kolatkar and Weis, 1996; Kolatkar et al., 1998; Zelensky and Gready, 2005; Lee et al., 2011; Sancho and Reis E Sousa, 2012).

Among the CLRs, Dectin-1, Dectin-2, MCL, Mincle, MR, and DC-specific ICAM3-grabbing non-integrin (DC-SIGN) have been implied in the recognition of fungal carbohydrates (Sancho and Reis E Sousa, 2012; Zhu et al., 2013). Dectin-1, Dectin-2, MCL, and Mincle employ the Immunoreceptor Tyrosine-based Activation Motifs (ITAM) to induce cell signaling through the activation of Src kinases, Syk, and PLCγ what leads to NF-κB and NFAT mediated transcription. Dectin-1 presents an intracellular tyrosine based motif named hemi-ITAM, since it presents only a LXXY, while a typical ITAM motif carries two similar separated tyrosine based motifs (YXXL/I). Dectin-2, Mincle, and MCL do not bear ITAM motifs, but they signal through the interaction with the ITAM containing protein, FcRγ chain. MR and DC-SIGN do not present ITAM motifs, although they are involved in the modulation of cytokine production by macrophages and dendritic cells and in the internalization of carbohydrate-carrying molecules and pathogens (Sancho and Reis E Sousa, 2012).

### Mannose receptor (CD206)

MR is a type-I transmembrane protein presenting an *N*-terminal cysteine rich domain, a fibronectin type II domain, eight extracellular CTLDs (1–8), and an intracellular portion that contains a motif involved in the endocytic signaling, FENTLY (Martinez-Pomares, 2012). MR recognizes glycoconjugates containing mannose, fucose, *N*-acetylglucosamine (Taylor et al., 1992; Taylor and Drickamer, 1993), sulfated *N*-acetylgalactosamine or sulfated galactose (Leteux et al., 2000; Liu et al., 2000). Recognition of sulfated polysaccharides is dependent on the cysteine-rich domain, but is independent of the CLTDs (Leteux et al., 2000; Liu et al., 2000). In contrast, recognition of mannose containing glycoconjugates requires the activity of the CTLDs 4–8, as demonstrated by binding assays employing recombinant versions of the MR CTLDs (Taylor et al., 1992).

MR is involved in the recognition of several fungal pathogens, such as *Pneumocystis carinii* and *C. albicans* (Ezekowitz et al., 1991; Netea et al., 2006; Cambi et al., 2008). MR has been described as a receptor involved in the phagocytosis of fungal pathogens (Ezekowitz et al., 1991; Cambi et al., 2008), but its role as a professional phagocytic receptor has been questioned when MR is expressed in non-phagocytic cells (Le Cabec et al., 2005). Although the ability of MR in triggering phagocytic signaling pathways has been put in check, it is well-established that MR promotes the endocytosis of mannose-containing ligands and participates in the phagocytosis of fungal pathogens (Ezekowitz et al., 1991; Burgdorf et al., 2006; Cambi et al., 2008). Thus, it is possible that MR acts as a receptor involved in the binding of mannose containing ligands what would permit the internalization mediated by another phagocytic receptor. Alternatively the phagocytic activity of MR must be a cell type specific property.

MR is also involved in the induction of signaling pathways that promote cytokine production in response to fungal pathogens and mannans (Netea et al., 2006; Tachado et al., 2007; Van De Veerdonk et al., 2009). TNF release by macrophages in response to *C. albicans* requires the recognition mediated by MR and TLR4 for *N*-linked mannans and *O*-linked mannans, respectively (Netea et al., 2006). MR has been demonstrated to recognize mannosylated glycoconjugates, such as phosphatydil-myo-inositol mannosides (PIMs) and mannose-capped lipoarabinomannan (LAM) from mycobacteria, and *O*-linked recombinant proteins expressed in *Pichia pastoris*. Thus, while *N*-linked mannoproteins seems to be the major ligands for MR in *C. albicans*, it is possible that other mannosylated glycoconjugates could be recognized by MR in fungal pathogens (Kang et al., 2005; Torrelles et al., 2006; Lam et al., 2007). MR cooperates with TLR2 in the induction of cytokines in response to *P. jirovecci*, and upon the *P. jirovecci* stimulation these receptors physically interact (Tachado et al., 2007). How MR contributes to signaling pathways involved in cytokine production is still unknown. MR has been described to promote the PPAR_γ_ expression and activation and this pathway is required for the induction of cytokines by mycobacterial ManLAM (Rajaram et al., 2010). However, MR has a short intracellular domain that lacks known signaling motifs involved in the gene expression of cytokines. Thus, it seems probable that MR works in cooperation with other receptors which are able to trigger the expression of cytokines, such as TLRs and CLRs.

### Dectin-2

Dectin-2 was initially characterized as a CLR expressed in a cell line derived from Langerhans cells. Dectin-2 is a type II transmembrane protein with one CLTD present in the COOH-terminal region and a short cytoplasmic tail. Dectin-2 activation is able to induce the production of cytokines and eicosanoids (Sato et al., 2006; Barrett et al., 2009; Saijo et al., 2010). Dectin-2 is expressed in macrophages, some populations of dendritic cells and in IL-6/IL-23 stimulated neutrophils (Ariizumi et al., 2000a; Taylor et al., 2005, 2014; Barrett et al., 2009; Robinson et al., 2009).

Dectin-2 has been described to recognize α-mannans (Saijo et al., 2010). Dectin-2 has an EPN motif in the extracellular domain, which has been demonstrated to be involved in the binding to mannose/fucose containing glycoconjugates (Ariizumi et al., 2000a). Dectin-2 binds to zymosan in a Ca^+2^ dependent mechanism that is inhibited by mannose, fucose, and in higher concentrations, *N*-acetylglucosamine, glucose, and galactose. Dectin-2 binds efficiently to extensively mannosylated synthetic carbohydrates, such as Man_9_GlcNac_2_, while binding decreases deeply with the reduction in the mannose residues (McGreal et al., 2006). Binding assays have demonstrated the Dectin-2 binding to BSA conjugated to different monosaccharides. Dectin-2 shows maximal binding to mannosylated and fucosylated-BSA, while the binding to BSA conjugated to *N*-acetylglucosamine, glucose or *N*-acetylgalactosamine is greatly reduced (Lee et al., 2011). Dectin-2 is a receptor involved in the recognition of *Malassezia spp* and *C. albicans*, and the Dectin-2 ligands have demonstrated to be a glycoprotein containing *O*-linked α-1,2-mannobiose residues, for *Malassezia* (Ishikawa et al., 2013), and α-mannans, for *C. albicans* (Saijo et al., 2010; Zhu et al., 2013).

Recently, Dectin-2 has been demonstrated to cooperate with MCL for the recognition of fungal mannans. Dectin-2 and MCL bind to mannans, and also form heterodimeric complexes. Although the expression of each receptor can promote the recognition of *C. albicans* hyphae and mannans, their association confers a higher sensitivity to the recognition of mannans and *C. albicans* (Zhu et al., 2013). Thus, the cooperation between MCL and Dectin-2 must represent a general mechanism of interaction of CLRs, extending and amplifying leukocyte responses to fungal carbohydrates.

### Dectin-1

Dectin-1 was first identified by means of the isolation of mRNA selectively expressed in a Langerhans cell derived line (Ariizumi et al., 2000b). Dectin-1 is a type II transmembrane protein and its structure comprises one CTLD in the extracellular portion, and a cytoplasmic region that presents the signaling motif, hemi-ITAM (Sancho and Reis E Sousa, 2012). Dectin-1 is expressed in macrophages, dendritic cells, neutrophils, and eosinophils (Brown et al., 2002; Taylor et al., 2002; Willment et al., 2005). Dectin-1 mediated signaling promotes cytokine production (Brown et al., 2003; Rogers et al., 2005; Rosas et al., 2008; Goodridge et al., 2009a), generation of ROS (Gantner et al., 2003; Kennedy et al., 2007), phagocytosis (Brown and Gordon, 2001; Brown et al., 2002) and dendritic cell maturation (Yoshitomi et al., 2005). Thus, Dectin-1 acts as a PRR connecting the recognition of fungal exposed β-glucans to the leukocyte activation and adaptive immunity induction.

Differently from many CLRs, Dectin-1 binding to β-glucans does not require Ca^+2^, although it occurs by means of the interaction of the extracellular CTLD with β1,3-glucans (Brown and Gordon, 2001; Adams et al., 2008). Surface plasmon resonance binding assays have demonstrated that Dectin-1 shows an extraordinary specificity to β1,3-glucans containing β1,6-branches, in contrast Dectin-1 do not bind mannans, pullulans, β1,6-glucans or β1,3/β1,4-glucans (Adams et al., 2008). The minimal structure recognized by Dectin-1 is a β1,3-heptasaccharide of glucose with a terminal β1,6-glucose branch, and higher polymerization degrees increase the affinity of Dectin-1 by β1,3-glucose oligosaccharides (Adams et al., 2008).

The mechanism of activation of Dectin-1 involves the clustering of the receptor by aggregates of β-glucans (Rosas et al., 2008; Goodridge et al., 2011). Although Dectin-1 shows a high affinity for soluble β-glucans, for example glucan phosphate presents an IC50 for the competition of the Dectin-1 binding to glucans of about 2 pmol L^−1^ (Adams et al., 2008), soluble β-glucans act as antagonists of the activation induced by β-glucan particles (Brown et al., 2003; Gantner et al., 2003). The activation of Dectin-1 requires its own clustering by β-glucan particles and the exclusion of the tyrosine phosphatases CD45 and CD148. CD45 and CD148 play a dual role in the Dectin-1 signaling, promoting the basal activity of Src kinases through the remotion of an inhibitory phosphotyrosine, while upon the clustering of Dectin-1/β-glucan particles, they become excluded from the signaling cluster, what must permit the ITAM signaling pathway to proceed (Goodridge et al., 2011). Thus, the activation of Dectin-1 is regulated by the physical nature of its ligands, with β-glucan particles of 0.5 μm or larger being strong activators of Dectin-1 (Goodridge et al., 2011).

Dectin-1 mediated responses are specific for some myeloid cells. Bone marrow derived macrophages and elicited peritoneal macrophages do not present Dectin-1 mediated responses, in contrast, alveolar macrophages, resident peritoneal macrophages and dendritic cells show Dectin-1 dependent responses to β-glucans (Rosas et al., 2008; Goodridge et al., 2009a). GM-CSF and IFN-γ can promote Dectin-1 responsiveness to non-responding cells, such as bone marrow macrophages, indicating that the responses mediated by Dectin-1 are flexible, according the program of differentiation of myeloid cells (Rosas et al., 2008; Goodridge et al., 2009a).

### Mincle

Mincle was firstly identified as a macrophage expressed gene dependent on the activity of the transcriptional factor NF-IL6 (Matsumoto et al., 1999). Mincle is a type II transmembrane protein containing a short intracellular tail and a CLTD in the extracellular domain. Mincle induces cell signaling through the interaction with the FcRγ chain which contains ITAM motifs and thus promotes Syk activation, and the NF-κB and NFAT mediated transcription of cytokines (Yamasaki et al., 2008).

Mincle has been demonstrated to be involved in the fungal recognition (Bugarcic et al., 2008; Wells et al., 2008; Yamasaki et al., 2009). Soluble recombinant Mincle binds to *C. albicans* (Bugarcic et al., 2008). Mincle is required for the TNF production by macrophages in response to *C. albicans* and *Clec4e*^−/−^ mice show a deficient clearance of *C. albicans* in an experimental model of infection (Wells et al., 2008). Mincle also recognizes the human commensal fungi, *Malassezia spp*. Mincle is able to confer NFAT activation by cell lines in response to *Malassezia spp*, besides *Clec4e*^−/−^ macrophages shows impaired cytokine production in response to *Malassezia spp* stimulation. Supporting the *in vitro* data, *Clec4e*^−/−^ mice show impaired leukocyte recruitment and cytokine production in a model of peritonitis induced by *Malassezia* challenge (Yamasaki et al., 2009). Recently, Ishikawa et al. have demonstrated that two *Malassezia* derived glycolipids are ligands for Mincle, a glyceroglycolipid containing the disaccharide gentiobiose joined to a glycerol backbone, which is acylated with C14 and C18 fatty acids, and a polar glycolipid composed by two mannosyl-10-hydroxy-octadecanoic acids and one dimannosyl-10-hydroxy-octadecanoic acid which are esterified to a mannitol core (Ishikawa et al., 2013). Although the ligands for Mincle have not been identified in other fungi, it seems reasonable that Mincle must recognize fungal glycolipids in other fungal pathogens, for example *C. albicans* (Bugarcic et al., 2008; Wells et al., 2008).

### DC-SIGN (CD209)

DC-SIGN is a type II transmembrane protein and its extracellular domain carries one CRD in the COOH-terminal portion and seven repeats which form an extracellular stalk that has been implied in the oligomerization of DC-SIGN (Geijtenbeek et al., 2000; Mitchell et al., 2001). The DC-SIGN CRD presents an EPN motif, and as expected DC-SIGN binds mannose-containing glycoconjugates, as well fucosylated carbohydrates, such as Lewis antigens, in a Ca^+2^ dependent mechanism (Appelmelk et al., 2003; Guo et al., 2004). The intracellular amino-terminal domain bears motifs involved in the internalization, such as triacidic and di-leucine sequences and non-ITAM/ITIM tyrosine based motifs (Van Kooyk and Geijtenbeek, 2003). DC-SIGN is an endocytic receptor involved in the binding and internalization of many pathogens, and it is expressed by dendritic cells and macrophages (Kwon et al., 2002; Geijtenbeek et al., 2003; Tailleux et al., 2003; Tassaneetrithep et al., 2003), as well as mannosylated and fucosylated antigens (Appelmelk et al., 2003; Frison et al., 2003; Guo et al., 2004). Coherently with its role as an endocytic receptor, the DC-SIGN mediated binding is decreased in the low pH values found during endosomal acidification, what indicates that, upon internalization by DC-SIGN, the cargo is released in the endosomal vesicles (Guo et al., 2004). DC-SIGN mediated recognition of mannose containing ligands has been described to amplify the cytokine production induced by TLR activation, while fucosylated ligands inhibit the induction of pro-inflammatory cytokines, but amplify the expression of IL-10 (Gringhuis et al., 2009). Furthermore, DC-SIGN activation by mannosylated lipoarabinomannans (ManLAM) has been demonstrated to inhibit the dendritic cell maturation by LPS (Geijtenbeek et al., 2003). Thus, DC-SIGN triggering has complex effects, promoting the entry of pathogens, inducing the production of cytokines, particularly IL-10, while inhibiting dendritic maturation, and it is speculated that DC-SIGN targeting by some pathogens could promote immune evasion during the infection of dendritic cells (Van Kooyk and Geijtenbeek, 2003).

DC-SIGN is involved in the fungal recognition. Expression of DC-SIGN in non-phagocytic cells is able to promote binding and phagocytosis of *C. albicans*. Besides, DC-SIGN is recruited to the dendritic cell phagosomes containing *C. albicans*, and its blockade decreases the *C. albicans* binding and internalization (Cambi et al., 2003, 2008). *N*-linked mannans have been described as the glycoconjugates responsible for *C. albicans* binding and internalization by dendritic cells, since a decreased binding of *C. albicans* is observed with deficient *N*-linked mannosylation strains (Cambi et al., 2008). In contrast, dendritic cells do not show any deficiency in the binding of *C. albicans* strains showing deficient *O*-linked mannosylation, lacking phosphomannans or terminal β1,2-mannoses. Interestingly, DC-SIGN mediated binding of *C. albicans* requires *N*-linked mannans, while intact *O*-linked mannans, phosphomannans or terminal β1,2 mannosides are dispensable. Although DC-SIGN is clearly involved in the recognition of *C. albicans* by dendritic cells, it cooperates with MR that makes the greatest contribution for the *C. albicans* binding to dendritic cells (Cambi et al., 2008). Altogether, these results indicate that MR and DC-SIGN mediate the recognition of *C. albicans N*-linked mannans promoting binding and internalization. DC-SIGN is also a receptor for the recognition of *A. fumigatus* conidia by human dendritic cells and macrophages. In contrast to *C. albicans*, DC-SIGN, but not MR receptor, is required for the binding of *A. fumigatus* conidia by human dendritic cells. *A. fumigatus* recognition by DC-SIGN is inhibited by purified mannans and galactomannans, and since *A. fumigatus* presents galactomannans as major mannose containing glycoconjugates, it must be an important target for DC-SIGN recognition by *A. fumigatus* (Serrano-Gómez et al., 2004).

The structural determinants for the DC-SIGN binding are the presence of terminal mannose or fucose residues. Binding assays have demonstrated that the soluble DC-SIGN CRD binds to mannans, mannose-containing oligosaccharides, and Lewis antigen structures, in a Ca^+2^ dependent mechanism (Appelmelk et al., 2003; Guo et al., 2004; Van Liempt et al., 2004). DC-SIGN binding to mannosylated proteins is inhibited by soluble fucose- and mannose-conjugated proteins, and in a lower extension by glucose-conjugates, while *N*-acetylgalactosamine- and *N*-acetylglucosamine-conjugates are ineffective as competitors (Lee et al., 2011). Crystallographic analyses demonstrate that mannose and fucose residues interact with the primary binding site in the CRD domain, in coordination with Ca^+2^ that interacts with two hydroxyl groups, while adjacent residues, such as galactose or mannose, present, respectively in high mannose containing structures, interact with secondary binding sites (Guo et al., 2004). Recognition of fucosylated Lewis structures requires strict interaction of a galactose with the secondary binding site, and the sialylation of the adjacent galactose abolishes the DC-SIGN binding to Lewis^X^ and Lewis^A^ ligands while sulfation reduces the binding (Appelmelk et al., 2003; Guo et al., 2004).

Although DC-SIGN has been pointed as a receptor involved in the binding and internalization of fungal pathogens, the roles of DC-SIGN in modulating dendritic cell and macrophage responses to fungi are still unknown. DC-SIGN has been implied in the induction of IL-10 and inhibition of dendritic cell maturation, so it would be interesting to evaluate the impact of DC-SIGN mediated recognition in the responses of dendritic cells and induction of T cell mediated responses to fungal pathogens. Alternatively, DC-SIGN could cooperate with other PRR, amplifying the pro-inflammatory induction in response to fungi, as previously observed with LAMs and TLR agonists.

### CD11b/CD18 (MAC-1, CR3)

CD11b/CD18 is a member of the leukocyte specific integrins that share the β_2_ common chain, also identified as CD18. CD11b/CD18 is a heterodimeric complex composed of the non-covalently associated type I proteins, the αM chain (CD11b) and the common chain CD18. It is expressed by leukocytes, including neutrophils, monocytes, macrophages, eosinophils, and NK cells (Ross, 2000; Hynes, 2002).

Besides its roles as a mediator of the leukocyte adhesion to the activated endothelium and the phagocytic receptor for iC3b opsonized particles (Holers, 2014), CD11b/CD18 has also been described to recognize β1,3-glucans. The αM chain presents two distinct domains involved in the recognition of the ligands by CD11b/CD18, the I-domain that is involved in the binding to iC3b, ICAM-1, and fibrinogen, while a distinct lectin domain has been implied in the recognition of β1,3-glucans, *N*-acetyl-*D*-glucosamine, glucose, and mannose (Thornton et al., 1996).

CD11b/CD18 has been demonstrated to be the major receptor involved in the binding of zymosan and *S. cerevisiae* yeasts, as well as the ROS production by these stimuli, by human neutrophils and macrophages, while Dectin-1 was demonstrated to be dispensable for these responses (Van Bruggen et al., 2009). Otherwise, the cooperation of Dectin-1 and CD11b/CD18 is necessary for neutrophil responses to zymosan and β-glucans, while macrophage recognition of β-glucans relies only in the Dectin-1 mediated recognition (Li et al., 2011). This mechanism requires the inside-out activation of CD11b/CD18 by Dectin-1 mediated recognition of β-glucans which promotes Vav1,3 activation and thus enables CD11b/CD18 binding and internalization of β-glucans by neutrophils, as well as ROS production (Li et al., 2011). In contrast with the recognition of β-glucan particles, the binding of soluble β-glucans by human monocytes and neutrophils has been demonstrated to be dependent on the β-glucan opsonization by iC3b which promotes CD11b/CD18 binding (Bose et al., 2013). Interestingly the responses of blood mononuclear cells to β-glucans particles requires the Dectin-1 mediated recognition, while soluble β-glucans require the CD11b/CD18 mediated detection (Bose et al., 2014). A similar role for Dectin-1 recognition of β-glucan particles, in contrast to a role for the complement for soluble β-glucans, has been observed for the antitumoral responses induced by the treatment with these β-glucans in murine experimental models (Qi et al., 2011).

While CD11b/CD18 has been convincingly demonstrated to be a receptor for β-glucans, there is still a considerable controversy about its role in the recognition of β-glucans, and this seems to extend in some experimental settings to the Dectin-1 mediated responses. Differences in the experimental settings must be responsible for the observed disparities, including (1) use of distinct β-glucan structures (particulated vs. soluble) (Rosas et al., 2008; Qi et al., 2011; Bose et al., 2013), heterogeneity and purity of the β-glucan particles, since zymosan and fungi are in fact highly heterogeneous and contains mannans, as well as chitin and lipids, furthermore zymosan is also a TLR activator (Van Bruggen et al., 2009), (3) presence of serum (a source of C3 that may trigger CD11b/CD18 activation even in situations where β-glucans were not recognized) (Bose et al., 2013), and (4) differences in the cell types investigated (neutrophils vs. macrophages) (Qi et al., 2011). Thus, CD11b/CD18 and Dectin-1 are receptors involved in the recognition of β-glucans and their activities must dictate the outcome of immune responses during the fungal infections. Besides that, while CR3 has been demonstrated to recognize fungal β-glucans, it has also been described to be a receptor for the internalization of mycobacterial glycoconjugate-coated beads, such as mycobacterial PIM_2_ or a succinylated glycopeptidolipid (Villeneuve et al., 2005). Thus, considering the ability that CR3 presents as a lectin receptor, it must also work as a receptor for fungal glycoconjugates other than β-glucans.

### CD14

CD14 is a glycosylphosphatidylinositol (GPI) anchored membrane protein presenting an extracellular amino-terminal portion containing leucine rich repetitions that assume a horseshoe-like conformation carrying a large hydrophobic pocket (Kim et al., 2005; Kelley et al., 2013; Zanoni and Granucci, 2013). CD14 has been demonstrated to be a receptor for bacterial LPS, and although CD14 lacks an intracellular signaling region, it cooperates with the TLR4/MD2 complex, conferring a highly sensitive recognition of LPS (Akashi et al., 2003). CD14 has been also described to work as a co-receptor for the recognition mediated by TLR2 (Schröder et al., 2004), TLR3 (Lee et al., 2006), TLR7 and TLR9 (Baumann et al., 2010).

CD14 is also involved in the recognition of fungal carbohydrates and glycoconjugates. Recognition of *C. albicans* and *S. cerevisiae* mannans is dependent on CD14, LBP, and TLR4 which shows similarities with the LPS detection, thus suggesting that LBP mediates the transfer of soluble mannans to CD14 and TLR4 (Tada et al., 2002). CD14 has also been demonstrated to be required for the recognition of fungal α-glucans (Bittencourt et al., 2006). Coherently with the observations of the CD14 mediated recognition of fungal carbohydrates, CD14 is also involved in the recognition of the fungi *A. fumigatus* (Wang et al., 2001; Mambula et al., 2002) and *P. boydii* (Figueiredo et al., 2010).

The structural bases of the CD14 interactions with fungal carbohydrates are still unknown. Crystallographic analyses of CD14 have revealed a NH_2_-terminal hydrophobic region buried in a horseshoe structure. Based on the proposed structure of CD14 and the structure of LPS, the large hydrophobic pocket seems to be site interacting with the lipid portion of LPS (Kim et al., 2005; Kelley et al., 2013). Otherwise, the hydrophilic O-antigen, a long and variable polysaccharide chain present in LPS, as well as peptidoglycan, have been described to interact with CD14 (Kitchens and Munford, 1995; Schwandner et al., 1999). The neighbor grooves and edges around the hydrophobic pocket are speculated to support hydrophilic interactions with polysaccharides (Kim et al., 2005; Kelley et al., 2013). Thus, although direct CD14 binding to mannans or α-glucans has not been evaluated, CD14 must be able to bind fungal polysaccharides using hydrophilic clefts. Based on the role of CD14 for the recognition of TLR ligands, it is probable that CD14 must bind fungal polysaccharides and load them into TLR4 or TLR2, thus promoting the signaling by these receptors.

## The recognition of chitin, a puzzle including several candidates and responses

While chitin and chitosan are clearly recognized by leukocytes, there is still a great controversy about the mechanisms of recognition of chitin. Silva et al. have demonstrated that chitin induces cytokine production by macrophages by a mechanism involving the recognition mediated by TLR2, MR, and Dectin-1 (Da Silva et al., 2009). In contrast, other studies have failed to demonstrate that highly purified chitin particles can induce pro-inflammatory cytokine production by peripheral mononuclear blood cells (Mora-Montes et al., 2011). Instead, *C. albicans* purified chitin has been demonstrated to inhibit the *C. albicans* induced cytokine production by blood mononuclear leukocytes (Mora-Montes et al., 2011). Highly purified chitin particles induce IL-10 production by macrophages and this requires the TLR9, MR, and NOD2 mediated recognition (Wagener et al., 2014). Chitin is able to reduce the inflammatory cell recruitment induced the *in vivo* LPS challenge. Corroborating the anti-inflammatory properties of chitin particles, a chitin deficient *C. albicans* strain induces an increased TNF release, what is mimicked by the inhibition of chitinases during the stimulation with the wild-type *C. albicans* strain (Wagener et al., 2014).

Many questions remain about the chitin recognition by leukocytes. The commonly used commercial sources of chitin have been demonstrated to carry contaminants of glucose, mannose, and undefined molecules what may contribute for some responses, such as cytokine induction by macrophages (Mora-Montes et al., 2011; Wagener et al., 2014). Furthermore, although macrophages are able to phagocytose chitin particles, the mechanisms involved are still unknown. The direct interaction of proposed receptors with chitin particles has also not been demonstrated. Even so TLR2 and TLR9 have been pointed as receptors for chitin induced cytokine release (Da Silva et al., 2009; Wagener et al., 2014), the eosinophil recruitment induced by chitin is MyD88 independent (Reese et al., 2007), thus excluding a role for TLR2 and TLR9 in the chitin induced eosinophilic inflammation. Besides, many aspects may influence the results obtained with chitin particles, such as (1) the presence of contaminants, (2) responses to distinct cell types (blood mononuclear cells, peritoneal macrophages, macrophage cell lines), (3) size and acetylation of chitin particles. Thus, although chitin represents a target for the innate immune recognition, more extensive analyses are required to characterize the immune responses to chitin and the receptors involved.

## Conclusions

**Figure 1:**
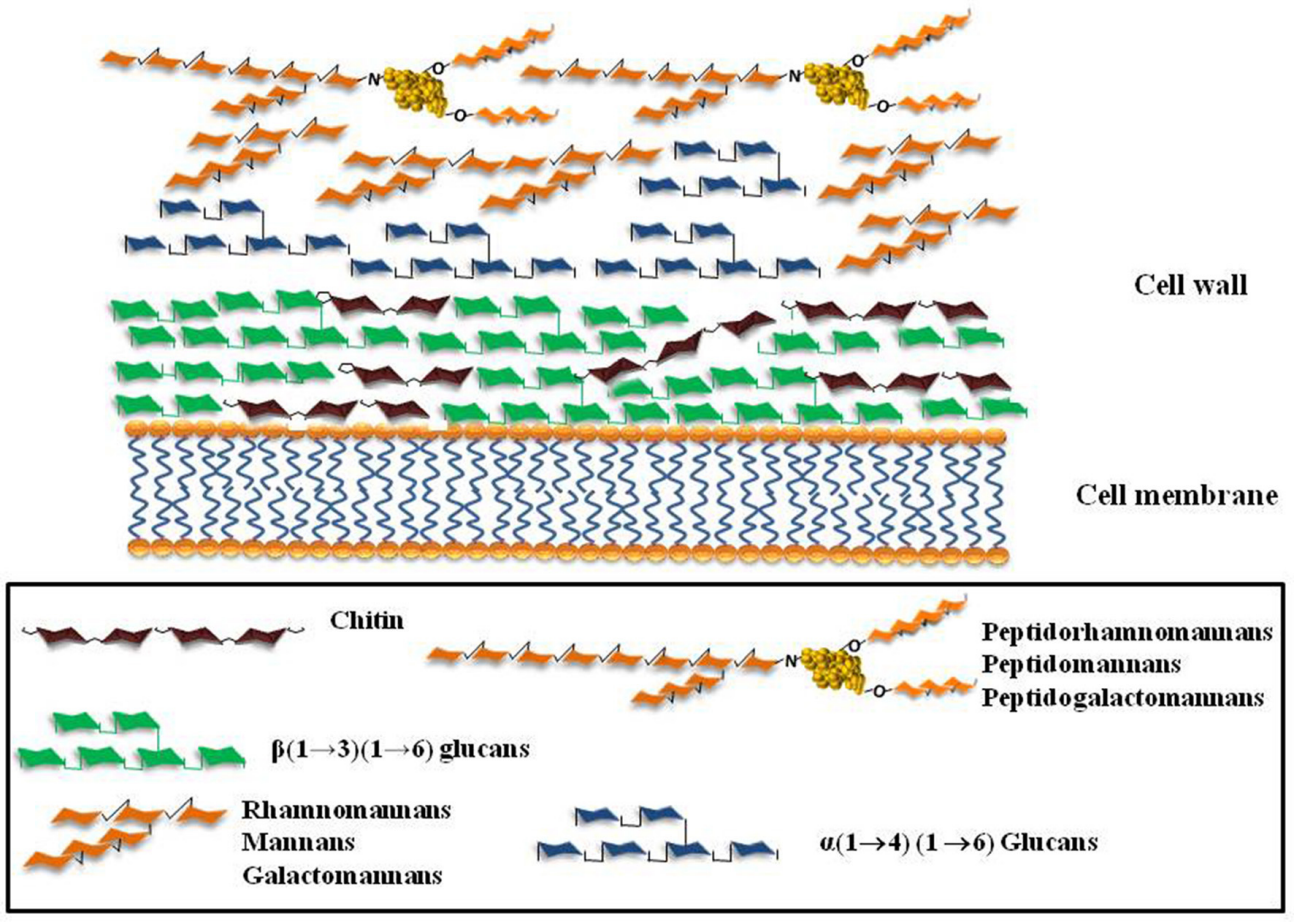
**Schematic representation of the major cell wall components of the fungi *A. fumigatus*, *C. albicans*, *Scedosporium/Pseudallescheria* complex**.

**Figure 2:**
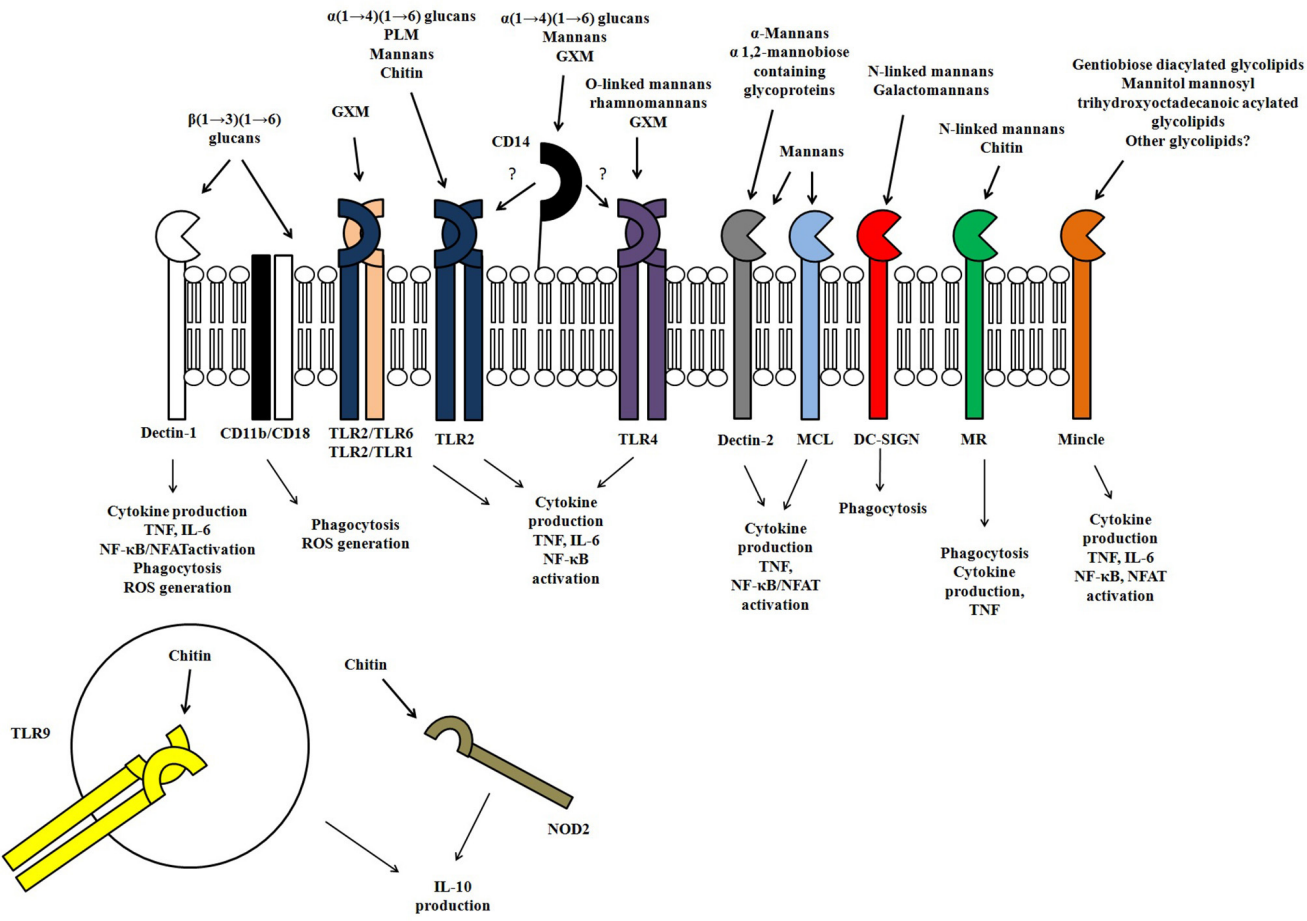
**Representation of the receptors involved in the innate immune recognition of fungal polysaccharides and glycoconjugates, as well the responses evoked in response to fungal polysaccharides**.

Polysaccharides and glycoconjugates are the major components of the fungal surface. Their structures vary among filamentous fungi and yeast and also among fungal species (Figure 1). The variety of carbohydrate structures present in the different fungal pathogens offers exceptional targets for the innate immune recognition which has evolved to recognize specific fungal glycans through a plethora of different receptors (Figure 2). Thus, the identification of fungal carbohydrates and their recognition by pattern recognition receptors must bring important contributions to the comprehension of the pathogenesis and immunity to fungal infections, and this must reveal new opportunities for the development of new classes of immunomodulators, antigens, and adjuvants.

### Conflict of interest statement

The authors declare that the research was conducted in the absence of any commercial or financial relationships that could be construed as a potential conflict of interest.
